# Immune suppression by neutrophils and granulocytic myeloid-derived suppressor cells: similarities and differences

**DOI:** 10.1007/s00018-013-1286-4

**Published:** 2013-02-20

**Authors:** Janesh Pillay, Tamar Tak, Vera M. Kamp, Leo Koenderman

**Affiliations:** 1 Department of Respiratory Medicine, University Medical Center Utrecht, HP. E 03.511, Heidelberglaan 100, 3584 CX Utrecht, The Netherlands; 2 Department of Anesthesiology, University Medical Center Utrecht, Heidelberglaan 100, 3584 CX Utrecht, The Netherlands

**Keywords:** Myeloid-derived suppressor cells, Neutrophil, Inflammation, Immune regulation, T-cell suppression

## Abstract

Neutrophils are essential effector cells in the host defense against invading pathogens. Recently, novel neutrophil functions have emerged in addition to their classical anti-microbial role. One of these functions is the suppression of T cell responses. In this respect, neutrophils share similarities with granulocytic myeloid-derived suppressor cells (G-MDSCs). In this review, we will discuss the similarities and differences between neutrophils and G-MDSCs. Various types of G-MDSCs have been described, ranging from immature to mature cells shaping the immune response by different immune suppressive mechanisms. However, all types of G-MDSCs share distinct features of neutrophils, such as surface markers and morphology. We propose that G-MDSCs are heterogeneous and represent novel phenotypes of neutrophils, capable of suppressing the immune response. In this review, we will attempt to clarify the differences and similarities between neutrophils and G-MDSCs and attempt to facilitate further research.

## Introduction

Neutrophils are important effector cells in the innate immune response against invading micro-organisms [1]. The cells possess multiple powerful mechanisms enabling them to migrate towards, engage with, in particular, small targets and kill them intracellularly [1]. The importance of these cells is illustrated by the fact that neutrophils and/or neutrophil-like cells have already developed early in evolution [2]. Cells with phagocytic function and neutrophil-specific proteins are now found in species ranging from simple organisms such as sea fan corrals [3] to complex organisms such as mammals [4].

The evolution from simple to complex organisms resulted in the origin of the adaptive immune system. This review will focus on recent data showing the existence of multiple functional phenotypes of neutrophils that, beyond their well-recognized anti-microbial functions, are able to steer and shape the adaptive immune system. But before reviewing these functional phenotypes in detail, it is important to first discuss recent data with respect to: (1) definitions for priming and phenotypes and (2) the life cycle and compartmentalization of neutrophils.

### Switching phenotype and priming: two distinct mechanisms

**Fig. 1:**
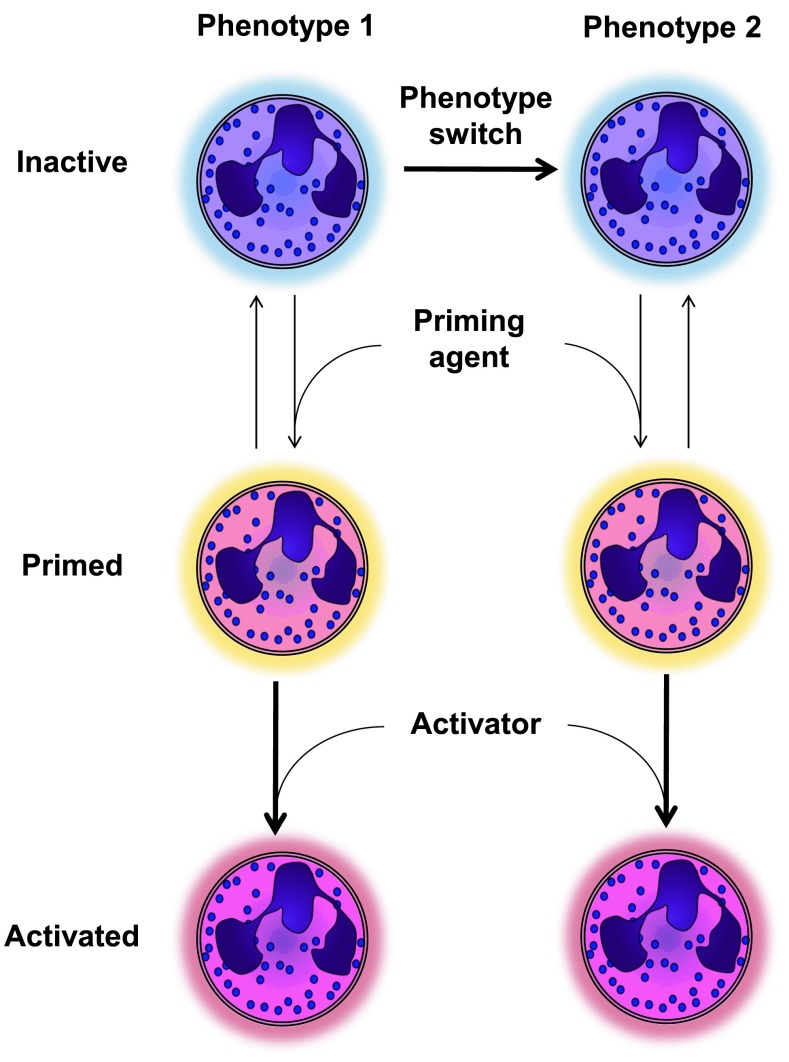
Priming versus functional phenotypes of neutrophils. This figure illustrates that phenotypes are defined as cells that retain specialized functions for a prolonged time. Priming refers to the mechanism that is rapidly and reversibly induced by soluble or cell associated mediators such as platelet activating factor (PAF) [7], which potentiate functions of neutrophils but do not change their overall function. Priming can potentiate all different phenotypes and functions, such as migration, production of ROS and phagocytosis

In this review, we define granulocytic myeloid-derived suppressor cells (G-MDSCs) as a phenotype of neutrophils. A phenotype refers to a cell that either in the bone marrow or by instruction in the periphery (Fig. 1) develops towards a cell with a specialized function, which distinguishes it from other cells. In the case of G-MDSCs, this would be their ability to suppress the adaptive immune response. It is only recently that neutrophils are accepted to have multiple phenotypes and, surprisingly, little is known regarding the occurrence and induction mechanisms of these neutrophil phenotypes. Few examples exist of neutrophils switching between phenotypes and it is unknown whether neutrophils with different phenotypes differentiate from specialized precursors (see also below, e.g., Fig. 4). Phenotype switching by neutrophils has recently been reported by the addition of granulocyte macrophage colony-stimulating factor (GM-CSF) to mature and immature murine bone marrow-derived neutrophils. These neutrophils acquired properties of dendritic cells such as antigen presentation but retained their anti-microbial properties [5].

In contrast to induction of phenotypes, priming can also modulate the functionality of neutrophils. Non-primed neutrophils are relatively refractory to activation, limiting aspecific activation. This process functions as a safe lock mechanism and has been extensively reviewed elsewhere [6, 7]. Only after priming (typically by a cytokine, chemokine or bioactive lipid) can a neutrophil optimally exert functions such as the generation of a respiratory burst induced by fMLF [8] or chemotaxis [9].

Priming is a mechanism distinct from changing of phenotype, as it reversibly potentiates effector functions of neutrophils but does not change their overall function.

### The life cycle of a neutrophil

Despite the consensus regarding the importance of neutrophils in host defense. surprisingly little is known about very basic characteristics of these cells in respect to their life cycle. As stated above, it is only recently that neutrophils are accepted to have multiple phenotypes. A possible reason that neutrophil subtypes were overlooked is the view that they are short-lived cells, which perform their duty and subsequently rapidly go into apoptosis in the tissue. This view is based on experiments labeling and tracing neutrophils with radioactive isotopes [10–13]. These experiments, which used ex vivo and potentially toxic labeling techniques, showed a peripheral blood half-life of only 7–25 h. Our recent paper using in vivo labeling with the stable isotope ^2^H suggests a half-life of 3.8 days [14]. This result remains a matter of debate, as Li et al. [15] suggested that the observed results could also be explained by a 3.8-day division time of neutrophil progenitors. Moreover, the view that neutrophils in tissue cannot return to the peripheral blood has been challenged by several studies. Already in 1974, Vincent et al. [16] showed in calves that, after disappearance of most labeled neutrophils from blood, hydrocortisone can induce their return into the circulation, where they stay for at least another 24 h. More recently, several studies have provided additional evidence that support the view that neutrophils do not simply die by apoptosis in the tissues but move to additional sites in the body. These studies show homing of neutrophils to secondary lymphoid tissue [17] and reverse migration of cells over endothelium in vitro and in vivo [18, 19]. Reverse migration and remobilization of neutrophils has also been shown very elegantly in zebrafish larvae demonstrating migration of neutrophils from a site of inflammation toward different organs throughout the organism [20].

Taken together, these data demonstrate that at least a subpopulation of neutrophils can survive for much longer than previously appreciated, allowing more time for these cells to switch phenotypes and exert functions beyond cytotoxicity against invading pathogens.

## Myeloid-derived suppressor cells

One of the recently described neutrophil phenotypes is the myeloid-derived suppressor cells (MDSCs). These cells were firstly identified at the beginning of this century and described as immature myeloid cells that suppress immune responses in the spleens of tumor-bearing mice [21–23]. Such immune suppression was earlier attributed to myeloid cells, but this activity was confined to differentiated cells such as macrophages [24]. As research progressed on these immature myeloid cells, it became clear that they consisted of a heterogeneous group of cells, consisting of (precursors of) granulocytes and monocytes, and that these cells were not always immature [25]. The term myeloid-derived suppressor cell was coined in 2007 by Gabrilovic et al. [26] to encompass the heterogeneity of these cells.

Considering the granulocytic component of MDSCs, there is still discussion on their differences and similarities with neutrophils. Recently, research on neutrophils described various novel neutrophil functions, such as antigen presentation, inhibition of immune responses, and induction of B cell class switching [27–29]. In addition, it has been known for decades that neutrophils reside in the spleen in health and disease [30], a location frequently sampled for MDSCs [31–33]. As the research fields concerning neutrophils and granulocytic MDSCs seem to have evolved in separate ways, this review will attempt to clarify the differences and similarities between these cells and attempt to unify and guide further research.

## Identification of neutrophils and G-MDSCs

G-MDSCs are MDSCs of *granulocytic* origin. According to this definition, these cells can belong to one of three different types of granulocytes: neutrophils, eosinophils, and basophils. However, only neutrophils have been described as a component of MDSCs [34, 35]. Multiple surface markers and characteristics that identify G-MDSCs have been described. Before going into detail about the different G-MDSCs characteristics, we will first clearly define how to identify a neutrophil in order to discuss the similarities and differences with G-MDSCs.

### Neutrophil identification

**Fig. 2:**
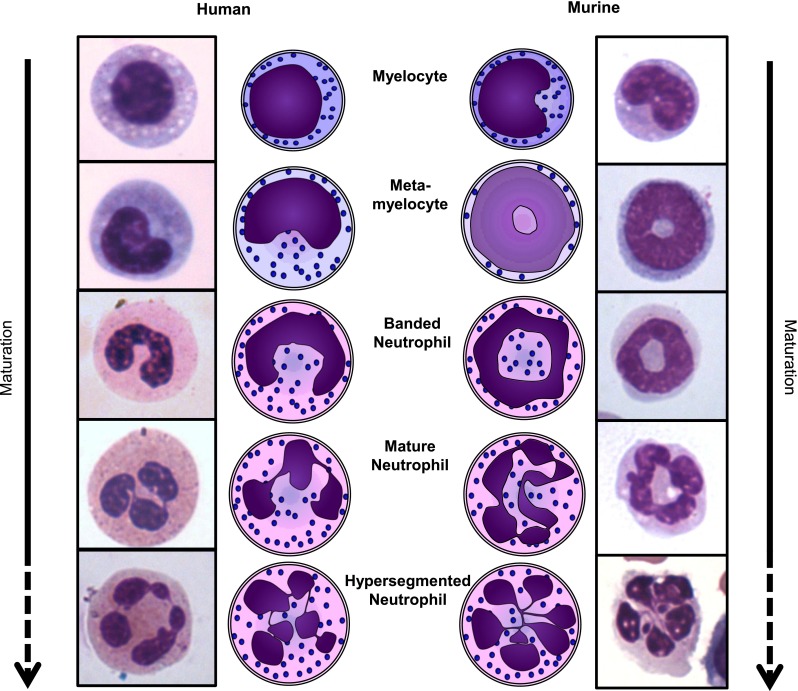
Schematic representations and images of the nuclear morphology of human and murine neutrophils during subsequent stages of development. Myelocytes mature into metamyelocytes, banded neutrophils, and finally into mature segmented neutrophils. Neutrophils may also become hypersegmented, with more than 4 nuclear lobes (human) or a cloverleaf shape (mouse). It is unknown whether hypersegmented neutrophils are more mature than segmented neutrophils

The gold standard to identify a neutrophil is by visual inspection under a light microscope. When stained with May-Grünwald-Giemsa or similar, neutrophils can be easily distinguished by the shape of their nucleus and cytoplasmic color/granularity (Fig. 2). The nucleus should either have a band or (hyper)segmented shape and a light pink/purple cytoplasm filled with similarly colored (“neutrophilic”) granules [36].

Identification of neutrophils by flow cytometry may be more convenient than visual inspection, as the latter is a more laborious and subjective method. In mice, flow cytometric identification of neutrophils can easily be performed by using the neutrophil-specific marker Ly6G [37]. Traditionally, Ly6G is combined with CD11b, but this is not necessary when using the specific Ly6G antibody 1A8 [37].

Human neutrophils lack a marker similar to Ly6G, but can be reliably identified nonetheless (Table 1). In studies on MDSCs, CD11b and CD33 are traditionally used as markers for human MDSCs. However, these markers are expressed on all cells of the myelocytic lineage and on NKcells, so they are not specific enough to identify human neutrophils [38–40]. Other markers used are CD14 and CD15. Neutrophils (or G-MDSCs) are found to be CD14^neg/low^ and CD15^pos^, whereas monocytes (or Mo-MDSCs) are CD14^high^ and CD15^neg/low^ [35]. Unfortunately, these two markers are not sufficient to identify neutrophils, as eosinophils have a similar CD15 expression [41]. We suggest CD16 as an additional marker, as mature neutrophils are CD16^high^, eosinophils are CD16^neg^, and monocytes either CD16^neg^ or CD16^int^. Therefore, CD16 allows for distinction between these two types of granulocytes. An additional advantage of using CD16 is that its expression varies between the different stages of neutrophil maturation: neutrophil progenitors capable of dividing are CD16^neg^, with increasing expressions in metamyelocytes, banded and mature neutrophils, respectively [38]. CD16 alone is not enough to identify neutrophils, since NK cells and monocytes also express this marker [42].

**Table 1:** Expression of the markers commonly used to identify human neutrophils or G-MDSCs

	Neutrophil (mature)	Eosinophil	Monocyte	NK cell
CD14	±	−	++	−
CD15	++	++	±	−
CD16	++	−	+	++
CD11b	++	++	++	++
CD33	+	+	+	+

In short, we suggest the use of Ly6G for identification of murine neutrophils and the combination of CD14, CD15 and CD16 for identification of human mature neutrophils. We do want to emphasize the importance of visual inspection, which remains the gold standard to identify neutrophils. Visual inspection should routinely be performed in order to eliminate the possibility of other cell types expressing neutrophil markers under certain clinical conditions.

### G-MDSCs versus neutrophils

As mentioned above, G-MDSCs have been implicated to have a similar expression of CD14 and CD15 as neutrophils, while mature or banded G-MDSCs and neutrophils also have similar CD16 expression. However, there seems to be one prime feature that distinguishes them from normal neutrophils: immune suppression. Several methods have been proposed to distinguish between the suppressive G-MDSCs and circulating neutrophils and will be discussed below.

### Identification of G-MDSCs: flow cytometry

Several papers have shown differences between G-MDSCs and normal neutrophils in the expression of cell surface markers visualized by flow cytometry. Greifenberg et al. [43] identified two subsets of neutrophils with a different CD11b expression in the spleens of healthy mice. Of these two populations, only the relatively low (but still positive) CD11b-expressing cells were found to be immune suppressive and, therefore, exhibited characteristics of G-MDSCs. Youn et al. [44] found an increased proportion of neutrophils expressing SLAMF4 (CD244) in mice bearing several different tumors. In some, but not all, of these tumor models, there was also an increased population of neutrophils expressing CSF1-R (CD115). When they compared the CD244-positive and -negative populations, only the CD244^pos^ cells were found to be immune suppressive. The consequences of these findings for the human situation remain to be established.

In humans, an enhanced expression of the IL-4Rα (CD124) was found on suppressive cells. This marker was found on the G-MDSCs of patients with non-small cell lung carcinoma [45]. However, another paper found CD124 expression to correlate only with immune suppression by monocyte-derived MDSCs [46]. Therefore, it remains uncertain whether CD124 can be used to identify human G-MDSCs.

In severely injured patients and in a human acute inflammation model, our group has identified distinct neutrophil subsets of which the CD62L^dim^/CD16^bright^ subset was immune suppressive [29]. In contrast to the findings by Greifenberg, who showed G-MDSCs to be lower in CD11b expression, this CD62L^dim^/CD16^bright^ subset showed a trend of higher CD11b expression [43]. Other markers upregulated in these suppressive cells were CD11c, CD32, CD35, CD45, and CD66b. The suppressive cells could, however, not be clearly distinguished on the basis of these latter markers.

Puga et al. [28] show two different subtypes of neutrophils in the human spleen, named N_BH1_ and N_BH2_ (B cell helper neutrophils). These subtypes have a higher expression of B cell activating factor (BAFF) and CD11b, and lower expressions of CD15, CD16, CD62P, and CD62L compared to blood cells. Additionally, the N_BH2_ cells have a higher CD27, CD40L, CD86, and HLA-II compared to both circulating and N_BH1_ neutrophils. Unfortunately, they only assessed immune suppression by splenic neutrophils as a whole. Therefore, it is unclear whether only one of these two subtypes or both are suppressive and which markers can distinguish between suppressive and normal neutrophils.

In conclusion, many markers are shown to distinguish suppressive G-MDSCs from non-suppressive neutrophils. However, so far, none of these candidates have been confirmed by other papers and some findings are contradictory (e.g., CD11b, IL-4Rα). Thus, to date, no single or combined expression of surface markers can reliably identify suppressive neutrophils or G-MDSCs in either humans or mice.

### Identification of G-MDSCs: density centrifugation

Centrifugation of blood over a layer with a density of 1.077 g/ml is a common step in the isolation of leukocytes from whole blood [47]. Due to their relatively high density, neutrophils end up below the layer, on top of the erythrocyte fraction, whereas the PBMC fraction is found in the interphase between this layer and the plasma. Schmielau and Finn [48], and Rodriquez et al. [49] found immune suppressive G-MDSCs in the PBMC fraction of cancer patients. These cells show an activated phenotype, characterized by increased CD66b and CD11b expression. Also, they show the immune suppression to be mediated by the CD66b-expressing cells [49]. However, they did not show whether the neutrophils with normal density in the same patients were also suppressive, and therefore it remains uncertain whether density centrifugation can distinguish between suppressive and non-suppressive cells. In vitro activation of neutrophils from healthy donors resulted in neutrophils with a similar density and suppressive capabilities, indicating that, in this system, G-MDSCs might be activated neutrophils [49]. Density centrifugation remains a widely used method for the isolation of human MDSCs in cancer patients, but there is still a lack of data on the differences between these G-MDSCs and neutrophils from these patients [50].

### Identification of G-MDSCs: gene profiling

Even though it is not possible to isolate cells based on gene expression patterns, it is likely that cells with different functions will have different gene expression profiles. Fridlender et al. [51] showed differences in the transcriptome of naïve bone marrow neutrophils in healthy mice, blood G-MDSCs from tumor-bearing mice, and tumor-associated neutrophils (TANs). The cells from the blood of tumor-bearing mice have a low expression of mRNA for cytokines and chemokines compared to TANs. Compared to bone marrow cells, G-MDSCs show a low mRNA expression of granule proteins, NADPH complex subunits, and peroxidases. Unfortunately, the location of neutrophils can influence their functionality [52], so it is unclear whether these differences were specific for G-MDSCs or a result of different localization/maturation. For instance, it is likely that neutrophils produce their granule and respiratory burst proteins during maturation and store them for later use, explaining the high amounts of mRNA for these proteins [53, 54].

Another transcriptome analysis by Youn et al. [44] compared neutrophils from naïve and tumor-bearing mice. It showed an upregulation of MPO and proteins involved in cell-cycle pathways in G-MDSCs from tumor-bearing mice. In contrast, neutrophils from naïve mice show an upregulation in mRNA for cytokines, chemokines, proteases, and other pro-inflammatory proteins.

Other proteins found to be upregulated in G-MDSCs are arginase-I [49, 51, 55, 56], iNOS [57], and IL-10 [55]. As these three proteins are directly involved in mechanisms of immune suppression by G-MDSCs, they will be described in more detail in the section below.

### Identification of G-MDSCs: nuclear morphology

MDSCs are in general described as young or immature cells [58]. The nuclear morphology of neutrophils provides a simple tool to assess their age. Neutrophils possess a distinct nuclear morphology in different stages of development (Fig. 2). Early progenitors have a round nucleus, which changes during maturation into the horseshoe, or “banded”, shape of a human immature neutrophil (a ring-shape in mice). When these cells fully mature, the nucleus starts showing indentations and is called segmented. When the nucleus has 4 or more segments in humans, or a cloverleaf-shape in mice, it is called hypersegmented. Since neutrophils gain more indentations and segments upon maturation, it is tempting to address hypersegmented cells as “old”. However, there is evidence that segmented and hypersegmented neutrophils in humans are of similar age [59].

In the paper of Greifenberg et al. [43] mentioned above, the G-MDSCs population had a clear ring-shaped morphology, whereas the cells with a segmented nucleus were not suppressive. This supports the notion of G-MDSCs being young/immature cells. Also, Fridlender et al. showed in a tumor model that immune suppressive TANs are mostly immature, whereas, after TGF-β inhibition, the TANs were found to be hypersegmented and did not suppress tumor growth, thus implying loss of immune suppression [37].

Other papers, however, shave shown no difference in nuclear morphology for the suppressive cells [44, 60]. Similarly, Dumitru et al. [35] have extensively reviewed the phenotype of suppressive G-MDSCs in human cancers and found them to be segmented in 8 out of 9 papers where the nuclear morphology was assessed [45, 48, 49, 56, 61–64]. In addition, in our model of acute inflammation and in severely injured patients, we have shown only the hypersegmented cells to be immune suppressive [29].

Taken together, nuclear morphology is not a good indication for immune suppressive functions and, therefore, of G-MDSCs. However, these differences do indicate the existence of several distinct G-MDSCs subtypes.

### G-MDSCs identification and subtypes: conclusion

When studying potential G-MDSCs (or suppressive neutrophils), one should first ascertain the cells of interest to be neutrophils. This can be done by flow cytometric determination of CD14, CD15, and CD16 expression and, ideally, assessing nuclear morphology after cell sorting. Density centrifugation is not a suitable method for isolating suppressive neutrophils, as it cannot distinguish suppressive cells from non-suppressive activated cells.

In various studies, different surface markers are shown to distinguish G-MDSCs or suppressive neutrophils from their non-suppressive counterparts. However, there are differences in expression of (activation) markers and nuclear morphology between these suppressive subsets. This demonstrates that there are several G-MDSCs phenotypes, possibly reflecting differences in localization, clinical condition, or origin.

## Mechanisms of immune suppression by suppressive neutrophils and G-MDSCs and their relevance to disease

**Fig. 3:**
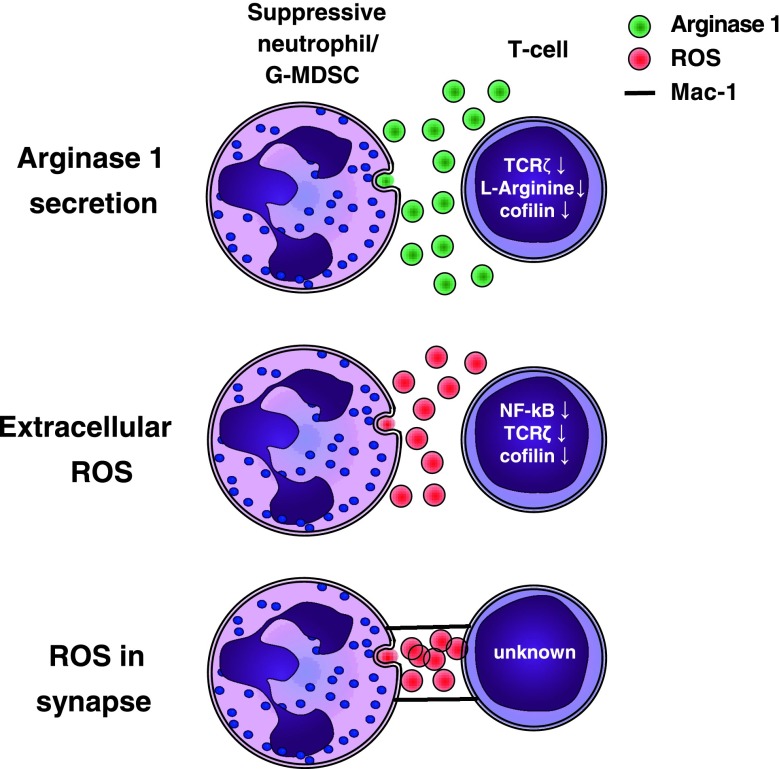
Mechanisms of suppression by G-MDSCs and suppressive neutrophils. Suppression can be mediated by extracellular arginase, extracellular ROS, or ROS in an immunological synapse. Al these mechanisms result in reduced T cell proliferation, via decreases in extracellular l-arginine, cofilin, TCRζ expression, NF-κB activation, or unknown mechanisms

Proliferation of T cells is influenced by many environmental factors. These factors, such as cytokines, growth factors, and amino acids, are easily altered in an inflammatory environment in the presence of other inflammatory cells such as neutrophils and G-MDSCs. Suppression of T cell responses can be achieved by depletion of essential amino acids from the microenvironment, such as l-arginine [65], (massive) generation of reactive oxygen species [48], or through cell–cell contact (Fig. 3) [29].

Production of anti-inflammatory cytokines such as IL-10 by neutrophils has been proposed [60, 66]. However, this was only observed in murine neutrophils [67] and will, therefore, not be discussed in this review.

Recently, studies have shown that, in addition to limiting T cell responses, G-MDSCs limit NK-cell responses and activation to *vaccinia* virus [68]. This was dependent on H_2_O_2_ production by G-MDSCs. Other studies have shown reduced NK-cell responses by G-MDSCs in pregnancy, cancer, and in the tumor environment; however, no mechanism of suppression was reported [69–71].

### The role of arginase in T cell suppression by MDSCs

Arginase-1 (ARG1) was shown to be important in the suppression of immune responses by MDSCs in various murine models [72]. ARG1 metabolizes l-arginine into l-ornithine and urea. This depletes l-arginine from the micro-environment. The amino acid l-arginine has multiple roles such as its importance in wound healing [73]. In addition, it is the only endogenous substrate for the production of nitric oxide (NO) by inducible nitric oxide synthase (iNOS) [74]. l-arginine is necessary for T cell proliferation, as, in the absence of l-arginine, the cell cycle of proliferating T cells arrests in the G0–G1 phase. [65].

Several mechanisms have been described to explain this l-arginine depletion mediated inhibition of proliferation. l-arginine influences the expression of the T cell receptor ζ chain (TCRζ, CD247) [75], and ARG-1 has been shown to downregulate TCRζ expression and T cell activation at the level of TCR expression [76, 77]. The TCR/CD3 expression is regulated by continuous internalization and recycling of receptors. The level of surface expression of the receptor regulates the ability of a T cell to become activated. The rate of synthesis of the TCRζ-chain is rate limiting to that of the other TCR/CD3 chains. Therefore, this chain is critically important in the regulation of TCR/CD3 internalization and recycling as it stabilizes the TCR/CD3 complex on the cell membrane. [78]. A second mechanism by which a depletion of l-arginine results in T cell suppression has recently been described. Feldemeyer et al. show that dephosphorylation of cofilin is decreased by depletion of l-arginine. Cofilin is a protein necessary for the remodeling of F-actin [79], which is essential for the formation of an immunological synapse and T cell proliferation [80].

ARG1 is widely expressed in murine myeloid cells and macrophages. However, in humans, it has only convincingly been shown in neutrophils [34, 81]. Neutrophil ARG1 is synthesized in their myelocyte and metamyelocyte stages and is located in the gelatinase containing granules of mature neutrophils [53, 81]. It is implicated in the host defense against fungi [81]. Activated neutrophils exocytose a form of ARG1 that is catalytically active at pH 9.5–10.5 [53, 82]. This ARG1 becomes active at a physiological pH of 7.5 only after cleavage by a co-factor. The co-factor responsible for this cleavage has not been identified, but it has been suggested to be located in azurophil neutrophil granules.

Release of ARG1 by neutrophils requires cellular activation and degranulation of both tertiary (gelatinase) and azurophllic granules. As stated above, human MDSCs have been shown to co-localize with PBMCs when isolated by density separation. Interestingly, fMLF-activated neutrophils from healthy volunteers show similar behavior and co-localize with PBMCs [48]. In patients suffering from severe traumatic injury, the increase of ARG1 activity has also been attributed to activated neutrophils in the PBMC fraction [83]. In addition, increased serum ARG1 correlates with degranulated neutrophils in patients with glioblastoma multiforme [63]. These findings could implicate that G-MDSCs in humans that inhibit T cell proliferation via an ARG1-mediated mechanism are simply activated granulocytes [49].

As described above, ARG1 expression in myeloid cells of mice and humans is essentially different [84]. Human studies have only correlated the degree and occurrence of ex vivo measured ARG1-mediated T cell suppression to disease progression. Murine studies mostly focused on the association of ex vivo T cells suppression and occurrence of MDSCs in the spleen. The direct contribution of MDSCs to in vivo T cell suppression in T cell-mediated diseases has remained largely uninvestigated, although, recently, ARG1 has been shown to limit graft versus host disease (GVHD) in mice. In this study, ARG1-expressing monocytic MDSCs were generated by ex vivo incubation with G-CSF, GM-CSF, and IL-13. Adoptive transfer of these ARG1-expressing cells or administration of pegylated-ARG1 limited pathology in this model [72].

### Reactive oxygen species

A hallmark of neutrophils and G-MDSCs is the potential to produce large amounts of reactive oxygen species (ROS). These are generated by the NADPH–oxidase complex in neutrophils. A detailed and schematic description of the generation of ROS is presented by Nathan and Ding [85]. Generation of superoxide anion (O2^−^) is the first oxygen radical produced. O2^−^ can be converted to two substances that have been shown to mediate lymphocyte suppression. Firstly, O2^−^ can react with NO, producing reactive nitrogen species such as peroxynitrite. NO is generated by inducible nitric oxide synthase (iNOS) using l-arginine as substrate, linking the generation of reactive nitrogen species to l-arginine metabolism as described above. Reactive nitrogen species are utilized in some models by monocytic MDSCs, but not by G-MDSCs and neutrophils, and will, therefore, not be discussed in this review [58].

The second substance formed from O2^−^ is H_2_O_2_ (hydrogen peroxide). H_2_O_2_ can be converted by myeloperoxidase to hypochlorous acid (HOCl^−^). H_2_O_2_ can suppress lymphocyte proliferation through various mechanisms by inducing apoptosis, decreasing Nf-κB activation, downregulating TCRζ, and oxidation of cofilin [86–88].

Cofilin remodeling of F-actin is essential for the T cell effector function. Oxidation of cofilin results in its loss of Ser3 phosphorylation [87]. Dephosphorylated cofilin is unable to mediate actin depolimerization, thus severely disturbing actin dynamics and impairing T cell activation [80]. Similar to l-arginine depletion, oxidative stress correlates with TCRζ expression, although the exact mechanism is not known. In addition, oxidative stress blocks Nf-κB activation leading to impaired T cell activation [88].

Of note is that regulatory T cells have been shown to be resistant to oxidative stress [89]. This suggests that regulatory T cells are less suppressed than other T cells, thus enhancing the overall suppressive effect of H_2_O_2_ in vivo.

Suppression of T cell activation and proliferation requires high concentrations of H_2_O_2_ [48, 87], which can be provided by the presence of large numbers of neutrophils at the site of T cell activation. This might be due to the fact that hydrogen peroxide is unstable and is rapidly converted to H_2_O and O_2_. Indeed, activated neutrophils or G-MDSCs in cancer patients have been shown to inhibit T cell responses in a H_2_O_2_-dependent manner [48].

The relevance of H_2_O_2_ in the context of G-MDSCs or neutrophil-mediated suppression is difficult to study in animal models. This is mainly due to the diverse biological functions of H_2_O_2_. Besides immune suppression, H_2_O_2_ and its metabolites are involved in bacterial killing [90]. In addition, it functions as a signaling molecule necessary for diverse cellular functions [90] including chemotaxis of immune cells. It has recently been shown that H_2_O_2_ is a potent inducer of chemotaxis of neutrophil-like immune cells in a model of tissue injury in zebrafish [91]. Hydrogen peroxide might, therefore, also indirectly contribute to microbial clearance by attracting immune cells and killing bacteria. These functions of H_2_O_2_ are indispensable in immune processes and, therefore, complicate the interpretation of studies targeting H_2_O_2_ to define its role in immune suppression by G-MDSCs.

Caution must be taken in interpreting ex vivo suppression of T cell proliferation mediated by H_2_O_2_. Manipulation and isolation of neutrophils and G-MDSCs might lead to cell priming and aberrant activation. Also, adhesion to plastic culture dishes might result in cellular activation, degranulation, and reactive oxygen species production resulting in vitro suppression of T cell responses [92]. Activation of large number neutrophils from healthy volunteers has been shown to suppress T cell responses ex vivo [87]. Therefore, at least two possibilities exist on how H_2_O_2_ results in immune suppression in vivo. Firstly, a general oxidative environment described by Klemke et al. in which ‘normal’ activated neutrophils mediate immune suppression. Secondly, as described below, small amounts of H_2_O_2_ can be delivered via the formation of an immunological synapse providing specific and direct suppression of T cell responses. It would be useful to distinguish between these two mechanisms in future studies concerning G-MDSCs and neutrophil suppression by H_2_O_2_.

### Immunological synapse formation, the requirement of cell-to-cell contact

The potency of the above-described suppressive mechanisms would be greatly enhanced by cell-to-cell contact and the formation of an immunological synapse. H_2_O_2_ has a short half-life and can be degraded by many endogenous anti-oxidants. Therefore, release into a synapse would potentiate and concentrate local concentrations of H_2_O_2_, H_2_O_2_ is produced in an immunological synapse between T cells and macrophages and dendritic cells during antigen presentation, and results in decreased lymphocyte activation [93, 94]. We have recently shown that a subset of neutrophils in human inflammation is capable of directly delivering H_2_O_2_ to the surface of lymphocytes and thereby limiting T cell activation and proliferation [29]. This contact was dependent on CD11b/CD18, an integrin abundantly expressed by the G-MDSCs in mice. However, in mice, no requirement of cell-to-cell contact suppression by G-MDSCs was found. A very recent study showed that, in patients with gastric cancer, G-MDSCs isolated from the tumor site suppressed T cells in a contact-dependent manner [95]. Regretfully, no experiments were performed in this latter study to further elucidate the suppressive mechanism.

## Distribution of neutrophils and G-MDSCs in lymphoid organs

In order to modulate the function and proliferation of T cells, neutrophils or G-MDSCs need to come in contact with or in close proximity to T cells [96]. T cell proliferation is normally considered to take place in secondary lymphoid organs such as lymph nodes and the spleen [97]. Recently, T cell proliferation has also been shown at the site of inflammation [98, 99]. In order to suppress these T cells, neutrophils will have to be present at these sites. Indeed, many studies show neutrophil homing to sites of T cell proliferation, which will be reviewed in the following section.

### Neutrophils in lymphoid organs

#### Spleen

Neutrophils are known to migrate to the spleen under both homeostatic and pathological conditions [30]. Reinfusion of ex vivo ^111^Indium-labeled neutrophils in healthy controls showed the majority of label in the bone marrow, spleen, and liver [30, 100]. These studies imply that considerable amounts of neutrophils rapidly home to the spleen after release from the bone marrow. In addition, in mice, about 10 % of reinfused radiolabeled neutrophils migrated towards the spleen, which was not influenced by the maturation status of neutrophils or inflammation [101]. It is important to emphasize that ex vivo manipulation of the cells could have induced subtle changes affecting their homing behavior in vivo [102].

In the spleen, under normal homeostatic conditions, neutrophils reside on the border of the red and white pulp [103, 104] and the marginal zone, whereas T cells are found in the white pulp [103]. Consequently, neutrophils should migrate to the white pulp in order to contact the T cells or vice versa. Neutrophil migration to the white pulp has been shown after intraperitoneal injection of LPS in mice. This was shown to be CD14-dependent [104]. Also, after surgical trauma, neutrophils were found to co-localize with T cells in the spleen [77]. These data demonstrate that neutrophils migrate towards the T cell zones of the spleen in acute systemic inflammation.

#### Lymph nodes

During inflammation, neutrophils are found to migrate to lymph nodes [17, 105–111]. Already in 1987, neutrophil trafficking from lung to draining lymph nodes was described in dogs [1]. In this study, fluorescent microspheres were instilled in the lung of dogs and phagocytosed by neutrophils and macrophages. After 40 h, almost half of the cells in the draining lymph node were neutrophils containing microspheres [105]. Also, in a more physiological model of antigen uptake [111], neutrophils can migrate to draining lymph nodes [17, 106]. Neutrophils were detected in lymph nodes during infections with *Mycobacterium bovis* [107], *Salmonella* [108], and different parasites [109–111]. In some of these models, neutrophils were shown to alter [17, 111] or even inhibit the inflammatory response [106, 110]. The route of migration toward the lymph nodes [107–111] was via the lymphatic system [17, 105–107, 109].

### Suppressive neutrophils and G-MDSCs in the spleen

Almost all studies regarding G-MDSCs in the literature were performed with Ly6G-positive cells isolated from the spleen [31–33]. However, not all of these Ly6G-positive neutrophils in the spleen can suppress T cells [43]. An influx of G-MDSCs into the spleen in mice has been seen both in acute and chronic inflammation such as cancer models [31], parasite infection (*Trypanosoma cruzi*) [32], and superantigen stimulation (*Staphylococcal enterotoxin*) [33]. Numbers of G-MDSCs were increased up to 10-fold 14 days after *Trypanosoma cruzi* infection [32]. During superantigen stimulation, suppressive neutrophils with highly segmented nuclei were sorted from the spleen [33]; these cells bear a resemblance to the hypersegmented CD16^bright^/CD62L^dim^ neutrophils that are found in the blood after LPS challenge [29].

Some cancer models increase hematopoiesis, resulting in increased cycling of hematopoietic stem cells and hematopoietic activity in the spleen [112]. Younos et al. showed by in vivo BrdU labeling that in tumor-bearing mice granulocytic proliferation mainly takes place in the spleen, whereas, in control mice, granulocytic cells predominantly proliferate in the bone marrow [113]. The CD3+ cells in this model proliferate less in the tumor-bearing mice, but, unfortunately, they do not show that this immune suppression is a direct effect of the spleen granulocytes. There were also no microscopic pictures of these cells to show their maturation stage [113].

### Suppressive neutrophils and G-MDSCs in the lymph nodes

Fewer data are available to show suppressive neutrophils or G-MDSCs in lymph nodes. Sepsis induced an influx of immature myelocytes capable of T cell suppression in lymph nodes. These cells could be detected 10–14 days after sepsis and remained present in the lymph nodes for at least 12 weeks after sepsis. Cytospins obtained during this study showed a heterogeneous group of cells consisting of both monocytic and granulocytic origin [114].

Vascular endothelial growth factor (VEGF) is able to induce MDSCs in cancer models and is a factor important for immune evasion in several cancer models [115]. Upon infusion of VEGF, myeloid cells, including neutrophils, were massively increased in lymph nodes [116]. Unfortunately, the capacity of these granulocytes to suppress T cells was not tested. Another indication that MDSCs can migrate to lymph nodes came from a study of Watanabe et al. [117]. They showed that proliferation of T cells in the lymph nodes of leukocyte-depleted mice was low when injected with spleen cells (containing both T cells and MDSCs) from tumor-bearing mice, compared to proliferation after injection with control mice spleen cells [117]. Proliferation was measured in vitro using cells isolated from lymph nodes. Unfortunately, this model did not discriminate between granulocytic and monocytic MDSCs, so further research is necessary to draw definite conclusions about the presence and importance of suppressive neutrophils in lymph nodes.

### T cell proliferation outside the lymphoid organs

T cell proliferation is not restricted to lymphoid organs, because T cell proliferation was also found, e.g., at sites of viral infection [98, 99, 118–121]. In influenza infection, proliferating T cells in the lungs contribute substantially to the total number of cytotoxic T-cells in the lung [98, 118]. Also, the persistence and reactivation of influenza-specific CD8+ memory T-cells can take place in mice without secondary lymphoid organs [119]. Similarly, CD8+ T cells proliferate outside the secondary lymphoid organs in a model of *Herpes simplex virus* (HSV) reactivation. In this model, infected sensory dorsal root ganglia (DRGs) are transplanted into naïve mice, inducing proliferation in the DRGs of both memory CD8+ T cells from graft [99] and newly recruited CD8+ T cells from the host [120]. Even further, in RSV infected mice, CD4+ memory T cells proliferate and differentiate in the lung, but not in the lymph nodes [121].

Taken together, this shows that T cells can proliferate at sites of viral infection, which is exactly the place where vast amounts of neutrophils are found [122, 123]. Therefore, although it may contribute, neutrophil migration towards the secondary lymphoid organs is not necessary to dampen the immune response.

## Origin of G-MDSCs and suppressive neutrophils

**Fig. 4:**
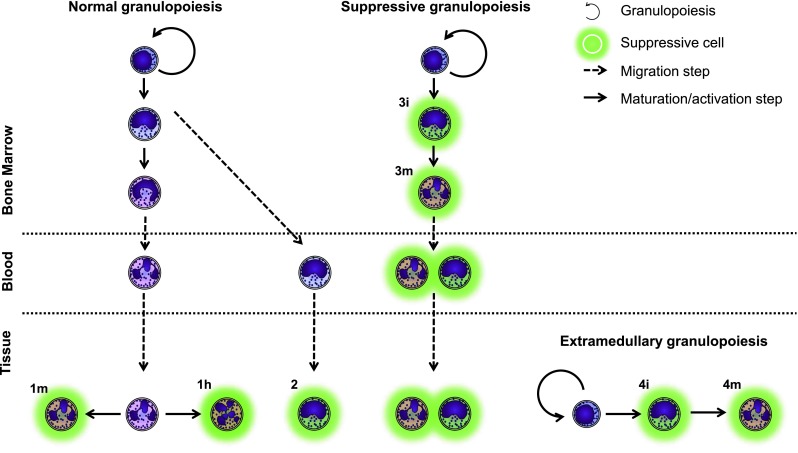
The origin of G-MDSCs remains unknown. Hypothetically, these calls can arise from mature (*1*) or immature (*2*) neutrophils receiving signals to become suppressive. Alternatively, there may be a dedicated granulopoiesis, which only produces suppressive cells. This granulopoiesis can take place either in the bone marrow (*3*) or extramedullary (*4*). Additionally, these cells can be immature (*i*), mature (*m*) or hypersegmented (*h*)

Many papers have shown only a subset of neutrophils to be suppressive. Even further, these suppressive subsets show differences in (flow cytometric) expression patterns and nuclear morphology [28, 29, 44–46]. The difference between normal neutrophils and the different types of suppressive neutrophils may lie in the presence of cytokines or growth factors, (e.g., G-CSF and VEGF) [115, 116, 124] in localization, or in their origin [28]. Few studies have addressed the origin of suppressive phenotypes, and therefore we will briefly discuss four hypotheses regarding the origin(s) of these suppressive cells (Fig. 4):

- Suppressive neutrophils might originate from normal, fully maturated cells. These cells acquire a suppressive phenotype under certain (inflammatory) conditions. They can either retain their mature nuclear morphology (Fig. 4, 1m) or become hypersegmented (Fig. 4, 1h).
- Cells do not fully mature before exiting from the bone marrow. Progenitors have been found in the peripheral blood under conditions of severe systemic inflammation caused by infection or trauma [125, 126]. These cells are neutrophil progenitors, which migrate to the tissue and subsequently become suppressive.
- An altered or a dedicated suppressive granulopoiesis, underlie the production of G-MDSCs, as suggested by the role of G-CSF in several papers [72, 124]. This results in either immature (Fig. 4, 3i) or mature (Fig. 4, 3m) cells with a suppressive phenotype.
- Instead of being produced in the bone marrow, suppressive cells might be produced by extramedullary granulopoiesis. This would result in either immature (Fig. 4, 4i) or mature (Fig. 4, 4m) cells with a suppressive phenotype. For example, Youn et al. [44] described G-MDSCs from tumor-bearing mice were produced in the spleen, whereas neutrophils from healthy mice originated from the bone marrow.

At this moment, it is unclear which of these mechanisms underlie the induction of G-MDSCs and whether multiple mechanisms co-exist. Further research is required to elucidate the origin of different suppressive phenotypes, and whether differences between suppressive phenotypes are caused by differences in their origin or by alternative activation.

## A novel hypothesis: G-MDSCs are a phenotype of neutrophils

Neutrophils do not belong to a single homogenous population of cytotoxic cells with a sole function to eliminate invading microorganisms. In fact, these cells can engage with and modulate T cells and, thereby, shape the adaptive immune system. The lack of consensus regarding nomenclature of these suppressive cells, their heterogeneity, and the lack of suppressive assays in many studies makes it difficult to draw overall conclusions. However, these studies support the hypothesis that multiple types of suppressive neutrophils exist, capable of mediating immune suppression by different mechanisms. Given the recent advances in neutrophil biology, illustrating their plasticity, we hypothesize that G-MDSCs might be a functional heterogenic subset of neutrophils. At this time, it is uncertain how many neutrophil phenotypes exist. It is, however, clear that targeting neutrophils or G-MDSCs as clinical intervention is only effective with knowledge of the different pro- and anti-inflammatory phenotypes, and when origin and kinetics of these cells are adequately elucidated.
